# Potential of Microfluidics and Lab-on-Chip Platforms to Improve Understanding of “*prion-like*” Protein Assembly and Behavior

**DOI:** 10.3389/fbioe.2020.570692

**Published:** 2020-09-08

**Authors:** Jose A. del Rio, Isidre Ferrer

**Affiliations:** ^1^Molecular and Cellular Neurobiotechnology, Institute for Bioengineering of Catalonia, Barcelona Institute of Science and Technology, Barcelona, Spain; ^2^Department of Cell Biology, Physiology and Immunology, Faculty of Biology, University of Barcelona, Barcelona, Spain; ^3^Center for Networked Biomedical Research on Neurodegenerative Diseases (Ciberned), Barcelona, Spain; ^4^Institute of Neuroscience, University of Barcelona, Barcelona, Spain; ^5^Department of Pathology and Experimental Therapeutics, University of Barcelona, Barcelona, Spain; ^6^Bellvitge University Hospital, Hospitalet de Llobregat, Barcelona, Spain; ^7^Bellvitge Biomedical Research Institute (IDIBELL), Hospitalet de Llobregat, Barcelona, Spain

**Keywords:** lab-on-chip, amyloid propagation, microfluidics, fibril, seeding, spreading, prion-like, prionoid

## Abstract

Human aging is accompanied by a relevant increase in age-associated chronic pathologies, including neurodegenerative and metabolic diseases. The appearance and evolution of numerous neurodegenerative diseases is paralleled by the appearance of intracellular and extracellular accumulation of misfolded proteins in affected brains. In addition, recent evidence suggests that most of these amyloid proteins can behave and propagate among neural cells similarly to infective prions. In order to improve understanding of the seeding and spreading processes of these “prion-like” amyloids, microfluidics and 3D lab-on-chip approaches have been developed as highly valuable tools. These techniques allow us to monitor changes in cellular and molecular processes responsible for amyloid seeding and cell spreading and their parallel effects in neural physiology. Their compatibility with new optical and biochemical techniques and their relative availability have increased interest in them and in their use in numerous laboratories. In addition, recent advances in stem cell research in combination with microfluidic platforms have opened new humanized *in vitro* models for myriad neurodegenerative diseases affecting different cellular targets of the vascular, muscular, and nervous systems, and glial cells. These new platforms help reduce the use of animal experimentation. They are more reproducible and represent a potential alternative to classical approaches to understanding neurodegeneration. In this review, we summarize recent progress in neurobiological research in “prion-like” protein using microfluidic and 3D lab-on-chip approaches. These approaches are driven by various fields, including chemistry, biochemistry, and cell biology, and they serve to facilitate the development of more precise human brain models for basic mechanistic studies of cell-to-cell interactions and drug discovery.

## Introduction

The formation of β-sheet enriched misfolded protein aggregates (also termed amyloids) via self-assembly of proteins or polypeptides, with intrinsic or induced amyloidogenic properties, is the hallmark of numerous protein misfolding diseases (PMD) affecting humans (Greenfield et al., 2008). These include neural diseases such as Alzheimer’s disease (AD), Parkinson’s disease (PD), multiple system atrophy (MSA), amyotrophic lateral sclerosis (ALS), and prionopathies [i.e., Creutzfeldt–Jakob disease (CJD)], as well as non-neural diseases such as pancreatic amyloidosis, systemic amyloidosis, and type II diabetes, among others (see Skinner et al., 2005; Ramirez-Alvarado et al., 2010; Sigurdsson et al., 2012; Otzen, 2013 for references). For several years, considerable effort has been made to uncover the molecular basis of the aggregation process and the different strains of specific amyloids with aggregative and infective properties. As a recent example of this interest, a published study of R. Nonno’s lab reveals that a small autocatalytic, but non-fibrillar, 7 kDa fragment of the pathogenic prion currently observed in Gerstmann-Sträussler-Scheinker disease (GSS) patients is also infective (Vanni et al., 2020). In fact, all these studies share as their goal the development of appropriate and specific methods to inhibit their aggregation in real biosafety and clinical scenarios (Giles et al., 2017).

Focusing on neuronal PMDs, several assays for amyloid detection, aggregation, and amplification have been developed in recent years to address the demand for reliable and sensitive *in vitro* detection of the various amyloid species (from oligomers to fibrils) in human samples. These procedures are mainly based on detecting the presence or monitoring the self-aggregation process of pathogenic amyloids using *in situ* or *ex situ* approaches. *In situ* assays are straightforward but they require direct introduction of probe molecules into the aggregation assay and in some cases interference in the process can be observed. Interference of the aggregation process by probe molecules can be avoided in *ex situ* assays, where small samples of an aggregating protein solution are extracted and diluted at specified time points into a buffered solution containing an appropriate dye molecule. Another alternative is to promote the amplification of very small quantities of the active pathological species up to significantly detectable levels, as the polymerase chain reaction (PCR) does for nucleotides. In the first group, available techniques range from classic fluorometric assays [i.e., Thioflavin T (ThT) (Saeed and Fine, 1967), Congo red (Yakupova et al., 2019)], and benzofuranone (for α-synuclein (Lengyel-Zhand et al., 2020), to new biosensor-based methods.

In addition, alternative classical optical detection methods for ThT staining (the most used fluorometric method) include the use of colorimetry techniques using, among others, gold nanoparticles (i.e., Zhou et al., 2015) or FRET methods on CdTe quantum dots (Xia et al., 2016).

Of these, numerous electrochemical biosensing methods based on antibodies {i.e., (Prabhulkar et al., 2012; Li et al., 2016), aptamers (Ylera et al., 2002; Zhao et al., 2020), coupling peptides (Li et al., 2013), and peptides with affinity regions [e.g., for β-amyloid the PrP(95-110)] (Rushworth et al., 2014)} were developed as reliable alternatives for conventional amyloid detection (see also Kaushik et al., 2016; Zhang et al., 2020 for review). These offer advantages compared to conventional methods due to their portability, relatively easy handling, and greater sensitivity. Among the huge number of emerging strategies, of particular interest is the application of new materials such as graphene oxide as a reliable alternative to fluorometric assays (e.g., ThT) to detect β-amyloid in label-free systems (e.g., Huang H. et al., 2017). Their use allows researcher to achieve simultaneous detection of oligomeric and fibrillar structures in the same sample (a challenging problem in classical fluorometric assays) and in other higher sensitivity studies, or the recently developed electrochemical determination of β-amyloid oligomers using a graphene and reduced graphene oxide dual-layer biosensor (Sethi et al., 2020). These are just some examples of the tremendous progress that has been made in the development of sensors for detection of amyloids (mainly β-amyloid). Readers interested in the emerging biosensors and electrochemical detection of amyloids may obtain more details in recent reviews (e.g., Serafin et al., 2020; Zhang et al., 2020).

A second strategy is amplification of the pathogenic amyloid in the presence of its monomeric counterparts using biochemical reactions, such as the protein misfolding cyclic amplification (PMCA) method (Saborio et al., 2001; Soto et al., 2002), real-time quaking-induced conversion (RT-QuIC) assay (Wilham et al., 2010; Sano et al., 2018), and ASA assay (Colby et al., 2007). These methods have evolved and have been adapted for the reliable detection of the presence and aggregative stage of various amyloids (i.e., Orru et al., 2017; Saijo et al., 2019) in different biological samples (i.e., Fairfoul et al., 2016; Haley et al., 2016; Bongianni et al., 2017). In parallel to these biochemical methods, other engineering laboratories have developed ultrasensitive methods based on graphene oxide and entropy-driven strand displacement reaction (ESDR) with LOD of 20 pM (Zhou et al., 2018), or graphene field-effect transistor (G-FET) for β-amyloid formation (Kuo et al., 2018). Unfortunately, these methods are not currently used in patient-derived studies.

**FIGURE 1:**
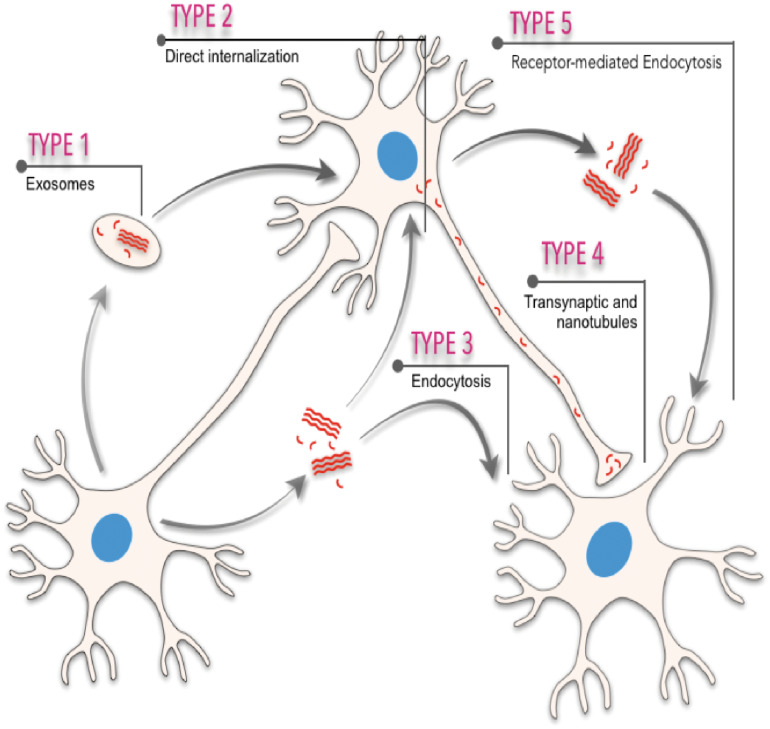
Scheme illustrating the different spreading pathways of a “hypothetical” pathogenic seed between different cells. Five different means of transmission are illustrated: (i) from exosomes or multivesicular bodies, (ii) direct internalization by interacting with plasma membranes, (iii) endocytosis or micropinocytosis processes, (iv) transsynaptic transmission and tunneling nanotubules, and (v) receptor-mediated uptake of the amyloid.

Neurodegenerative progression in PMDs runs in parallel with disease-specific characteristic intra- or extra-cellular accumulation of misfolded amyloids. In fact, cellular, molecular, biophysical, and biochemical studies have revealed that most PMDs are progressive disorders, and that amyloid-associated pathologies spread from diseased to healthy cells affecting different brain areas in a sequential basis (see Costanzo and Zurzolo, 2013; Goedert et al., 2017; Holmes and Diamond, 2017; Del Rio et al., 2018; Vilette et al., 2018; Meisl et al., 2020; Peng et al., 2020 for reviews). The spatiotemporal progression of these diseases seems to correlate, with some controversy (see below), with the brain propagation of the amyloid-associated neuropathology between anatomical pathways specific to each disorder, suggesting a cell-to-cell spreading of the disease (Saper et al., 1987; Bertrand et al., 2004; Braak and Del Tredici, 2009; Costanzo and Zurzolo, 2013; Goedert et al., 2017; Peng et al., 2020; Figure 1). However, this “connectome” view of the seeding and progression of the amyloid accumulation between connected areas is under debate for some PMDs [i.e., PD, please see Surmeier et al. (2017) for review]. In PD, when comparing the appearance and progression of the Lewy pathology in affected individuals with experimental mouse models of the disease, it seems to correlate better with cell- or region-autonomous mechanisms rather than connectivity (Surmeier et al., 2017). Nevertheless, several routes of inter-cellular amyloid spreading have been proposed such us membrane binding, receptor-mediated, and non-mediated endocytosis (i.e., Holmes et al., 2013; Mao et al., 2016; Aulic et al., 2017; Urrea et al., 2018; Rauch et al., 2020), multivesicular bodies (i.e., exosomes) (Sardar Sinha et al., 2018; Perez et al., 2019; Winston et al., 2019), or tunneling nanotubes (TNTs) (Costanzo and Zurzolo, 2013; Abounit et al., 2016; Zeinabad et al., 2016; Peng et al., 2020; Figure 1). Understanding the intercellular formation and transmission of these amyloids, also termed “pathogenic seeds” in some studies, has become a challenging issue in recent years (i.e., Vilette et al., 2018; Erana, 2019; Peng et al., 2020). Relevantly, the participation of neurons, astroglia, and oligodendrocytes also displaying insoluble amyloid inclusions in particular neurodegenerative diseases during seeding and spreading is still unknown and warrants further study (i.e., Kovacs et al., 2017; Ferrer, 2018).

In addition, considering the evidence demonstrating the ability of these amyloids to disseminate protein misfolding as “pathologic seeds” from sick to healthy cells, a “*prion-like*” or “*prionoid*” hypothesis has been proposed (Ashe and Aguzzi, 2013; Aguzzi and Lakkaraju, 2016; Collinge, 2016; Woerman et al., 2018). Indeed, cell-spread of amyloid seeds can act as a self-propagating template disrupting cell viability and leading to both the death of recipient cells and the progression of the neurodegenerative disorder (Ashe and Aguzzi, 2013; Aguzzi and Lakkaraju, 2016; Collinge, 2016; Holmes and Diamond, 2017). However, some criticisms appeared with this terminology [i.e., for tau (Polanco and Gotz, 2015), β-amyloid (Watts and Prusiner, 2018), and MSA-derived α-synuclein (Woerman et al., 2020)]. We suggest to the reader interested in this topic examining the reports of Castilla’s and Requena’s laboratories (i.e., Castilla and Requena, 2015; Erana et al., 2017) and the recent review of Wells et al. (2019). In fact, prion diseases (e.g., CJD) are the only neurodegenerative disorders showing an infectious transmissible protein species, the pathogenic prion, capable of recapitulating a clinical disease (e.g., Prusiner, 1998a,b; Moore et al., 2009). Moreover, the prion strain also plays a crucial role in dictating the type (i.e., punctate vs. plaques) and anatomical distribution of the lesions of pathogenic PrP deposition in affected brains (see for example Fraser and Dickinson, 1973; Lasmezas et al., 1996; Scialo et al., 2019 for a recent review).

In this challenging scenario, microfluidics and lab-on-chip (LOC) technologies have emerged in the last 15–20 years as a plausible strategy to monitor some aspects of amyloid aggregation and amyloid-biological interactions, as well as a being a valuable, reproducible tool to analyze cell-to-cell seeding and spreading of pathogenic seeds from “*prion-like*” or “*prionoid*” proteins (Aguzzi and Rajendran, 2009; Scheckel and Aguzzi, 2018). In fact, the use of microfluidics and LOC technology has extended from technical fields (i.e., engineering, physics, and electronics) into a wide variety of biomedical scientific fields including pharmacology, oncology, immunology, and neurobiology.

### An Overview of Microfluidics and Lab-on-Chip (LOC) Platforms

Microfluidics platforms are devices containing microchannels with a height/width scale between 100 nm and 100 μm (Tabeling, 2005; Folch i Folch, 2013). Although for neuroscience, pioneer microfluidic studies were developed by Campenot (1977, 1982), (see also Taylor and Jeon, 2010; Neto et al., 2016 for reviews), from the electronics point of view, its origins begin in parallel to microelectronics by micro-fabrication techniques from the semiconductor industry in the 1970s and 1980s (please see Tabeling, 2005). After this, in the 1990s the concept of μTAS (miniaturized total chemical analysis systems) was developed to describe a microfluidic platform that could carry out all the functions required for analysis of an analyte: sample preparation including transport, and chemical reactions as well as selective analyte detection. From this, in terms of manufacture, terms like MEMs (microelectromechanical systems) and LOC devices emerged in the microelectronics and biomedical fields (Folch i Folch, 2013). As indicated in the introduction, LOC might include biosensors/electrochemical/optochemical sensors that have been developed in recent years. In fact, there is a parallel development of the complexity of the LOC devices (i.e., in terms of manufacture) directed to amyloid screening and monitoring with the development of new detection methods.

Today, although with some exceptions (Kamande et al., 2019), the vast majority of microfluidic LOC devices utilized in neurobiology are generated using the silicone elastomer polydimethyl-siloxane (PDMS) (McDonald et al., 2000; McDonald and Whitesides, 2002; Ng et al., 2002; Kuncova-Kallio and Kallio, 2006). PDMS is an economically cheap, biocompatible, soft, flexible, and easy-to-handle elastomer, with an index of refraction of 1.43 (similar to a glass coverslip ≈1.52), and good gas diffusion. Relevant electrical and thermal isolation are highly suitable for biological research (i.e., cell culture) and fluorescent microscopy (McDonald et al., 2000; McDonald and Whitesides, 2002; Ng et al., 2002; Kuncova-Kallio and Kallio, 2006). This new PDMS-based microfluidic application using the elastomer directing microfluidic, micropatterning, and microfabrication technologies to neurobiological experiments was developed 2003–2006 by Jeon’s lab (Taylor et al., 2003, 2005; Rhee et al., 2005; Park et al., 2006) with great development in recent years (see Neto et al., 2016 for review). These pioneer studies follow the experiments of Campenot (1977, 1982) in generating a simple, reproducible, and tunable culture platform for compartmentalized neural growth and differentiation. Most of the experiments designed to explore the cell dynamics of different amyloids are currently based on their pioneer microfluidic designs.

PDMS-based manufacture of LOC devices is mainly based on “soft-lithography” protocols (McDonald et al., 2000; Whitesides et al., 2001). Today, PDMS manufacture has evolved to multilayer LOCs as well as more complex platforms. In fact, due to their physical characteristics, different PDMS microfluidic chips can be bounded using plasma reactions (Tran H. T. et al., 2014) to generate multilayered LOCs (i.e., Saar et al., 2016). In fact, some of these multilayered LOCs are used today to analyze amyloid formation *ex situ* (i.e., Saar et al., 2016). Additional methods ranging from hot-embossing lithography (Jeon et al., 2011) to 3D printing methods (Au et al., 2016) have been developed to generate new LOC devices. Readers may obtain more information about LOC platform manufacturing strategies in reference microfabrication books (Minteer, 2006; Herold and Rasooly, 2009; Lee and Sundararajan, 2010; Folch i Folch, 2013). In this review we will focus on the PDMS-derived LOC devices with the greatest impact on the study of amyloids associated with neurodegeneration.

### Why Use LOC Devices and Microfluidics in Amyloid-Related Studies?

Microfluidics and LOC devices hold a number of advantages for amyloid-related research at different levels: (1) most LOC devices reduce the use of reagents due to small reaction volumes; (2) some LOC devices can produce a large number of independent but repetitive compartments to allow protein-protein interactions (i.e., micro/nanodroplets, see below); (3) in most LOC platforms we have complete control over spatial and temporal parameters in the reaction conditions; (4) most LOC devices are compatible with several detection methods (i.e., optical or electrical sensors); and (5) some LOC devices allow for the differential culture of different cell types (i.e., neurons, astroglia, oligodendroglia, microglia, etc.) in the same platform in an interactive way – for example in: (i) microfluidically isolated chambers, (ii) cells growing on molecular or chemical gradients, (iii) cells growing under flows, and (iv) cells growing inside 3D structures [cellular aggregation (i.e., neurospheres, neurospheroids, and organoids) or biomaterial-derived scaffolds].

In the large bibliography focused on microfluidics with more than 3000 published reviews reported in Pubmed^TM^, we will focus in this review on first describing the use of LOC platforms to monitor amyloid seeding and aggregation, and second whether LOCs help researchers understand cell-to-cell amyloid spreading and how LOC devices can help us analyze amyloid effects in neural network physiology. However, in order to understand their potential, first we will broadly summarize the most basic physical concepts of microfluidics that play crucial roles in the design of some of the approaches and devices that will then be further described. In addition, some specific references are included for those readers interested in particular aspects of microfluidics.

### Some Basic Physical Concepts in Microfluidics

In biotechnology and bioengineering, microfluidics and LOC are present in various forms depending on the application and end use. The most usual form is single-phase microflows inside channels or capillary tubes. This is the typical case of a physiological buffer or a culture medium that contains a molecule or analyte that should be detected or analyzed using an LOC device. However, for different studies we need to use multiphase flows. This implies the use of two different non-miscible fluids (for example in micro/nanodroplet formation or cell encapsulation). However, in both single and multiphase flows, fluids are often moved by applying hydraulic pressure to the fluid inside the channel/s depending on the LOC design. In some cases, the applied force is a gravitational force but in other cases syringe pumps are used to move the fluid. In order to understand fluid dynamics in single-phase microflows inside small microchannels, first we need to focus our attention briefly on their behavior. In microfluidics one of the most frequently used key values is the Reynolds number (*Re*). This is a dimensionless number expressed as a function of the density of the fluid (*ρ*), the velocity of the fluid inside the channel (*u*), the length of the channel (*L*), and the dynamic viscosity of the fluid (η)

$$R\,e=\frac{\rho \,u\,L}{\eta }$$

At the microscale (as happens in most LOC devices) the *Re* number is very low and the fluids behave like a laminar non-turbulent fluid. In fact, very few microfluidic devices used turbulent flows (with *Re* values > 2,000), but a differential range of fluid flows can be used in a laminar regime. Those microflows that show an *Re* value <0.5 follow the multiparametric Navier-Stokes formulation (see Tabeling, 2005; Bruus, 2008 for details). In these cases, aqueous fluids are considered Newtonian fluids which belong to the category of incompressible, uniform, and viscous fluids. However, for micro/nanodroplet or digital microdroplet approaches (see below) the use of two fluids (water and oil) with different behaviors is typical. In these cases, the LOC device can use non-Newtonian fluids that do not follow the formulation below. We refer the reader to classical reference books for specific formulation for non-Newtonian fluids (i.e., Böhme, 1987; Bruus, 2008; Berthier and Silberzan, 2010).

In addition to the Reynolds number, the interfacial tension also plays a role. It states the elastic tendency of a fluid in a surface to contract the surface-air interface in order to reduce its free energy (Sackmann et al., 2014). Interfacial tension is relevant in two immiscible fluids. In this situation and at this scale, interfacial tension dominates the gravitational force (Sackmann et al., 2014). Another dimensionless number is the capillary number. This number compares surface tension forces with viscous forces. Where σ is the interfacial tension, *u* is the velocity and η is the viscosity.

$$C\,a=\frac{\eta \,u}{\sigma }$$

**FIGURE 2:**
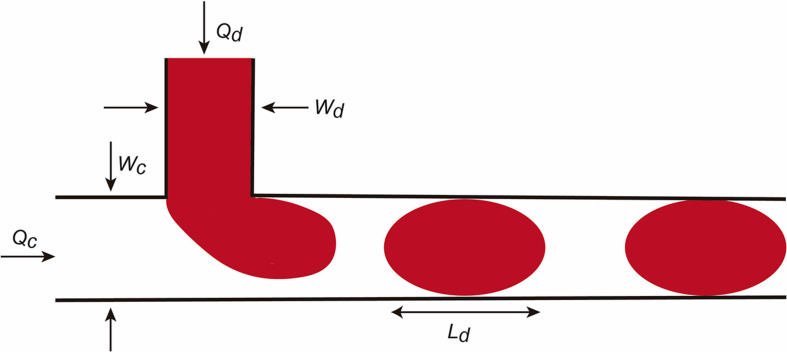
Scheme illustrating micro/nanodroplet formation in a classical T-junction LOC. Please see the main text for details concerning the *Q* and *W* values. In this configuration, the dispersed phase is pumped into the continuous phase orthogonally. As the dispersed phase enters the continuous, shear forces elongate the head of the dispersed phase until a segment eventually separates and relaxes into a sphere or plug shape as a result of interfacial tension.

In addition to these values, the Bond number or the Weber number are also described. We refer the reader to Tabeling (2005), Bruus (2008), and Grimmer and Wille (2020) for more details. The factors are relevant for droplet formation. In this review we will consider the droplet formation in a T-junction system in detail (Figure 2; Teh et al., 2008). The reader can obtain current information on droplet LOC designs in Grimmer and Wille (2020). In the scheme, the fluid flows are determined as *Qc* and *Qd* and the dimension of the channels *Wd* and *Wc.* In this LOC, the *Ca* is one of the most important parameters in droplet generation. For example, when the *Ca* number is low, the incoming dispersed phase fluid tends to occupy the full section of the channel and droplet formation occurs at the downstream side of the T-junction corner. However, if *Ca* is higher the dispersed phase fluid occupies only a part of the channel and smaller droplets are formed. Practically, when a droplet is generated it continues to grow for a time *t*_*n*_ for necking due to the continuous injection of the dispersed phase fluid. At the end, the final droplet volume *V* can be predicted (in an easiest vision) with this scaling law.

$$V=V\,c+t_{n}\,Q\,d$$

Where *Vc* depends on *Ca* and the duration of necking *t*_*n*_ and decreases as Ca increases. Another relevant factor in droplet formation is the flow rate radio *Q (Q* = *Qd/Qc)* (from dispersed and C for continuous phases). For example, for small *Q* the droplet is pinched off at the T-junction corner regardless of the *Ca* number. However, for large *Q*, increasing *Ca* will force the detachment point to move from the corner downstream. Due to the large surface-to-volume ratio, fluid/surface interactions largely affect droplet dynamics in the microchannels. In parallel, the presence of the droplet changes the hydrofluidic resistance of the microchannel. If fact, we can assume that the overall flow resistance of a channel can be estimated by:

$$R^{*}=R+n\,R\,d$$

Where *R* is the resistance of the channel, *n* is the number of droplets inside the channel, and *Rd* is the single droplet resistance. The value of *Rd* has been studied in several reports and is dependent of several factors such as the viscosity of the dispersed phase and the continuous phase, and the length of the droplet, as well as the geometry of the microchannel (see below). Considering these parameters today we can generate microdroplets for amyloid aggregation in micro/nanodroplets in a controlled space.

For non-bioengineering researchers, and considering Newtonian fluids, the third relevant aspect is that following microfluidic rules, we can correlate the behavior of a pressure-driven microfluidic laminar flow inside a channel with Ohm’s law of electricity (*R* = *V*/*I*). Thus, the ratio of the fluid pressure (*P*) ≈ *V* (voltage) and the volumetric fluid flow rate *Q* is the electrical current *I* (Figure 2). This occurs since under laminar flow, the pressure drop inside the microchannel is proportional to the flow rate: Δ*P* = *R*_Q_*Q. The hydraulic resistance *R*_*Q*_ (also termed *R*_*H*_ in some references) is dependent on microchannel geometry: the cross-section area (*s*) but also the length (*L*) of the microchannel. Thus, for an arbitrary section of the microchannel (with equal *x* and *y* dimensions) the value of *R*_*Q*_ may be expressed as follows:

$$R_{Q}=2\,\mu \,L\,\frac{p^{2}}{s^{2}}$$

Where *μ* is the dynamic viscosity of the fluid and *p* is the perimeter of the microchannel. Thus, in this situation the Hagen-Poiseuille equation can be used in a channel with circular section to determine the Δ*P* as follows (reviewed in Shah and London, 1978):

$$\Delta \,P=R_{Q}\,Q=\frac{8\,\mu \,L}{\pi \,r^{4}}\,Q$$

In the case of a channel with square section [height (*h*) = width (*w*)] or rectangular section (when *w/h* > 1), the hydraulic resistance of the channel (*R*_*Q*_) can be calculated respectively as follows (see Grimmer and Wille, 2020 for details):

$$R_{Q}=\frac{28.3\,\mu \,L}{h^{2}}\;\text{and}\;R_{Q}\approx \frac{a\,\mu \,L}{w\,h^{3}}$$

where *a* denotes (in rectangular section) a dimensionless parameter defined as

$$a=12\,{\left[1-\frac{192\,h}{\pi^{5}\,w}\,\tanh\left(\frac{\pi \,w}{2\,h}\right)\right]}^{-1}$$

However, in a presence of a droplet (see above) the resistance of the microchannel dynamically changes. In fact, the values for the *Rd* of the droplet are described by this formulation

$$R\,d=\left(\mu_{d}-\mu_{c\,o\,n\,t}\right)\,\frac{L_{d}\,a}{w\,h^{3}}$$

Where *L*_*d*_ is the length of the droplet, *μ_*d*_* the viscosity of the dispersed phase, and μ_*cont*_ the viscosity of the continuous phase and the other parameters described above. Consider that the Hagen-Poiseuille law is similar to Ohm’s law (see above). Thus, these calculations are very useful to modulate and determine flow, pressures, and channel geometry as well as to simulate the behavior of our microfluidic flows (i.e., culture media, droplet formation) inside a particular LOC. As examples, we will describe two different microfluidic devices with wide use in neurobiology using correlative microfluidics as well as electric circuit modeling (please see Oh et al., 2012 and Robertson et al., 2014 for additional details).

#### The Hydrodynamic Focusing/Mixing LOC Device

**FIGURE 3:**
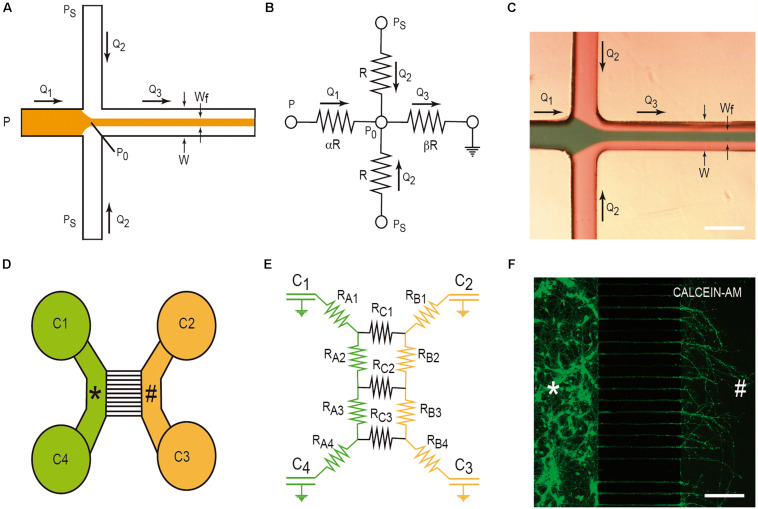
Examples of LOC devices and microfluidic platforms. Microfluidic hydrofluidic mixing device **(A–C)** and compartmentalized LOC device **(D–F)**. For each device, the correlative fluidic and electric model is included. In **(C,F)**, we provide two examples of the generation of hydrodynamic focusing **(C)** and the growth of axons labeled with Calcein^TM^. The two different compartments are labeled with * and # in **(D–F)**. Please see manuscript section “Some Basic Physical Concepts in Microfluidics” for details of the correlation between fluidics laws and electric laws in LOC devices.

Hydrodynamic focusing is a method to control continuous flow for solution mixing of fluids or droplets. The basis is the formation by confining fluid streams or flows to small geometries or microchannels. The main advantage of this strategy is the achievement of very short complete mixing times while maintaining a highly controllable system which can be precisely modeled. In the basic system (Figure 3A), there are three inlet streams: one central stream to be focused and two streams that converge perpendicularly on the central stream (Figures 3A–C). In the example, we consider, in Figure 3B, the electric model in which the section of all channels is identical (α) and the value β is the distance between the reference point P_0_ and the end of the mixing channel. Following Kirchhoff’s two laws, we can approximately define the *Wf* size in the mixing channel by applying the following equation based in fluid dynamics:

$$\frac{W\,f}{W}=\frac{1}{g\,\left(\lambda \right)}\,\frac{Q\,1}{Q\,1+Q\,2+Q\,3}$$

where *g* (*λ*) is a factor depending on the aspect ratio of the channel *h/W*, *h* being the height and *W* the weight of the microchannel, if *h* <<< *W*; *λ* −> 0, but more commonly *h* <1 and the formula is more complex (please see Berthier and Silberzan, 2006; Rodriguez-Villarreal et al., 2010 for details). Alternatively, and as first described by Knight et al. (1998), considering Ohm’s law and the electric model (Figure 3B), the summary of the calculation is the following:

$$r=\frac{P\,s}{P}=\frac{\beta \,Q\,1+\left(2\,\beta +1\right)\,Q\,2}{\left(\beta +\alpha \right)\,Q\,1+2\,\beta \,Q\,2}\,\text{and}\,W\,f\,=\frac{1+2\,\beta -2\,\beta \,r}{1+2\,\alpha \,r}\,$$

Thus, using these parameters we can control the interaction between two different fluids by modulating *Q1* and *Q2*. In fact, this was used in a large number of experiments (see Kirby, 2010; Badilescu and Packirisamy, 2011 for reference books). In a laminar flow regime mixing only occurs by diffusion. Thus, the mixing of the analytes depends only on their intrinsic diffusion coefficient (see Arter et al., 2020 for a recent review). After this, the diffusion parameters of the different elements (i.e., oligomers) can be controlled and analyzed during amyloid aggregation. In fact, these systems can also include several detection systems such as electrodes (stimulating or recording) as well as optical measurement. In this respect, the Peclét number (Pe) describes the relative rates of molecular convection relative to diffusion. Classically, the LOCs retain large values of Pe to prevent complete diffusional mixing over the timescale. Thus, LOCs are well suited to the study of protein-protein interactions. Typically, this is achieved through quantification of changes in the size or charge of proteins and complexes as they participate in the interaction. Due to the above-mentioned basis, the mixing rate of analytes under microfluidic flow can be measured by analyzing their diffusion coefficient and the hydrodynamic ratios of the biomolecules. With these approaches, the diffusion of α-synuclein (Zhang Y. et al., 2016) and β-amyloid (Scheidt et al., 2019) were analyzed. However, and as an alternative, these experiments have also been developed in droplets from fento to nanoliter volumes. These droplets can be generated at different frequencies (from 0.1 to 1 MHz). In contrast to bulk-phase studies in which aggregation reactions are dominated by secondary effects that rapidly amplify the rate of protein misfolding, masking primary nucleation events (i.e., Knowles et al., 2009), in LOCs droplets single nucleation events can be observed on a drop-by-drop basis, an approach that also enables observation of the spatial and temporal propagation of some fibrillar proteins (e.g., insulin; Knowles et al., 2011; Pfammatter et al., 2017).

#### Compartmentalized Cell Culture With Microchannels Growing Under Microfluidic Isolation in Two or More Chambers

This microfluidic device was developed to culture two or more different cell types (Figure 3D). In fact, their original goal was to compartmentalize cultured neurons in order to microfluidically isolate the somato-dendritic domain from the axonal domain for axonal RNA isolation for axotomy purposes (Figure 3D). This was achieved by using four open chambers with two channels that were interconnected by a variable number of microchannels (up to ∼100). These microchannels showed (in the original model) a small rectangular section with a high fluidic resistance due to its length. This largely reduced the fluidic flow between the two chambers. A more detailed description of the original microfluidic device can be found in Taylor et al. (2005). In order to isolate axons, although depending on the cultured neuron, the length of the microchannels should be more than 400–450 μm since dendrites or immature neurites are able to enter inside microchannels, but for cortical neurons, they only extend for a distance ≈150–250 μm in contrast to growing axons that might extend for distances longer than 1 mm inside the microchannels (Taylor et al., 2005; Figure 3F). Taking into account Ohm’s law, Robertson et al. (2014) developed an electric model of the device (Figures 3D,E). With this, we can model the volumetric transport of the media between the different chambers. In the modeled LOC device, the platform contains a network of resistances R_*A*__–C_, linking four chambers C_1_-C_4_ (currently of 7–8 mm ∅). Considering the volume of each chamber (acting as an electronic capacitator), the hydrodynamic pressures (electrical voltage) and the volumetric flow rates (electric current) can be predicted over time as follows:

$$V\,\left(t\right)=\frac{\Delta \,V}{2}\,\left(1-e^{-\frac{2\,t}{R\,C}}\right)\;\text{and}\;P\,\mathrm{(t)}=\,R\,\frac{d\,V}{d\,t}$$

where the flow rate *V(t)* is the differential volume between wells over time and *P(t)* is the differential hydrostatic pressure between wells over time. Depending on the values of *C* and *R* and the initial conditions of volume of each chamber, the time constant *t* = *RC*/2 provides us data to estimate the rate of change of the hydraulic pressure, and consequently allows us to estimate the time needed to reach a volume equilibrium in the chambers of the LOC device. In addition, this procedure is a very useful tool during device design, empowering optimization of the relationship between the assay to be performed and design of the microchannel geometry (i.e., length, cross-sectional dimensions, etc.) (Robertson et al., 2014). On a practical basis, we can modulate the volume of each chamber to generate small but controlled media flows that will impair, in parallel with the fluidic resistance inside the small microchannels, the unwanted diffusion of a putative analyte or amyloid through the medium based on a differential concentration. This will be very useful to determine cell-to-cell prion/amyloid transmission; furthermore, we can determine whether an amyloid is taken up by specific domains of the healthy or unhealthy neuron or glial cell (see below). From the numerical data evaluation, the authors analyzed two putative configurations (flow paths): a pressure gradient between wells connecting a culture chamber (i.e., C2 and C3) and a pressure gradient across the microchannels. Using these numerical simulation, the estimated time constants for the pressure to equilibrate were 189 s across each chamber (*t*_*B*_) and 39,956 s across the microchannels (*t*_*C*_), respectively (Robertson et al., 2014). As *t*_*C*_ is two orders of magnitude greater than *t*_*B*_, the two chambers can be considered fluidically isolated over short periods of time. However, the authors do not provide additional data concerning longer times. This is usually solved by increasing the volume of the culture medium in one part of the device with respect to the other. Another relevant aspect in these LOCs is the maintenance and survival of cultured cells. In our experience, we were unable to maintain cultured neurons for more than 20 days. This impairs a putative long-term experiment on infectivity and only specific processes can be analyzed. Furthermore, this limits the use of induced pluripotent stem cells (IPSc), with large differentiation protocols, in these devices. In order to avoid this, pre-differentiated neurospheroids are currently used in similar LOCs and some of these are commercially available (Osaki et al., 2020).

## Amyloid Aggregation Studies in LOC Devices

Although most amyloid species were initially identified within the context of neurodegenerative diseases and their transmission (see Peng et al., 2020 for a recent review), several proteins have also been found to form amyloid-like fibrils [e.g., islet amyloid polypeptide (amylin) (Zheng et al., 2020) or insulin (Zheng et al., 2020)] outside the nervous system. Furthermore, increasing evidence has pointed toward amyloid formation being a generic self-aggregation property of proteins and polypeptide chains, with many polymeric species having been identified during the process (see Morris et al., 2009 for review). In fact, a key aspect in evaluating the aggregation kinetics of biomolecular species is the ability to monitor fibrillar growth as a function of time (see above). The monitoring of this process can be developed in LOC [e.g., in hydrodynamic focusing systems (i.e., Fitzpatrick et al., 2013; Arosio et al., 2016)], or using electrophoretic approaches in these systems (i.e., Saar et al., 2018).

**FIGURE 4:**
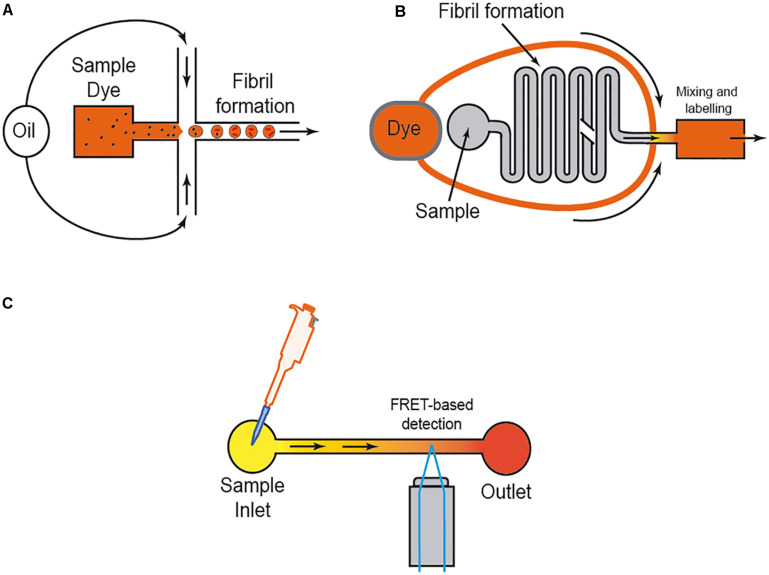
Scheme of three different approaches to analyzing amyloid formation under fluidic flow. In **(A)**, the micro/nanodroplet formation (water-oil) is illustrated. In **(B)** a mixing LOC device is illustrated, and in **(C)** the use of FRET derived protein sensors to monitor protein-protein interaction is illustrated. In each example, the presence of the dye (usually ThT) is displayed.

However, it is well-known that the amyloid aggregation process is largely dependent on the interaction of the protein with several ions (i.e., Kim et al., 2018; Metrick et al., 2019), membranes (i.e., Terakawa et al., 2018; Alghrably et al., 2019), and other surfaces (e.g., water; Schladitz et al., 1999). Thus, researchers used different methods to avoid or control these in some cases unwanted interactions. One of the most widely used methods is the aggregation study inside micelles or micro/nanodroplets (water-oil) of different proteins (i.e., Shim et al., 2007; Teh et al., 2008; Casadevall i Solvas et al., 2012; Shembekar et al., 2016; Figure 4). Using microdroplets, many independent reactions can be monitored simultaneously in identical volumes which are several orders of magnitude smaller than what has been common in biochemical assays. On a practical level, the formation of the droplets, mainly by microfluidic focusing devices, is followed by their harvesting and maintenance, trapped or retained in reaction chambers to be further analyzed (i.e., Shim et al., 2007; Teh et al., 2008; Casadevall i Solvas et al., 2012; Shembekar et al., 2016; Figure 4B). In these cases (especially for sessile droplets exposed to air) (Casadevall i Solvas et al., 2012), the decrease in droplet volume over time due to the evaporation of water molecules increases the analyte/protein concentration inside the generated droplet, thereby increasing protein-protein interaction and, more relevantly, amplifying signal(s) associated with the aggregation that can be monitored with several methods, including Förster resonance energy transfer (FRET) (Figure 4). Other alternatives to enhance the process of reduction of the microdroplet volume using PDMS-derived microflows to enhance the reactions were also recently reported (i.e., DroMiCo; Kopp et al., 2020). This recently published method generates and traps the microdroplets, the aqueous flow rate is stopped in the LOC, and the oil flow rate is kept minimal at 2 μL/min to accelerate droplet shrinking and prevent entry of air into the device, in order to further analysis.

In fact, amyloid fibril formation typically displays sigmoidal growth kinetics (i.e., Ferrone et al., 1985; Knowles et al., 2009; Morris et al., 2009; Prusiner, 2017). Indeed, changes in the aggregation of fibrils make up a three-phase curve that can be monitored easily using ThT-fluorescence or, depending on the protein sequence, intrinsic amino-acidic residues (i.e., tryptophan) with particular fluorescence properties after aggregation (i.e., Knowles et al., 2009; Meisl et al., 2016; Toprakcioglu et al., 2019). In current aggregation kinetics, the first stage is a “lag-phase” also termed “nucleation-phase” displaying a very small increase in the amount of fluorescence over time (i.e., Arosio et al., 2015), followed by a fast-growing phase and the last phase or “steady state” equilibrium with higher fluorescence values. However, we should consider that this is a dynamic process that includes fibrillar fragmentation, nucleation, and other processes during all the phases. In fact, the duration of the “lag-phase” is highly dependent not only on the monomer concentration and seed formation, but also on physical parameters as well as the presence of molecular chaperones. For example, when sarkosyl-insoluble fractions from AD patients or other tauopathies are analyzed in bulk experiments, the lag-phase cannot be clearly distinguished due to the high concentration of preformed fibrils and “amyloid seeds” (i.e., Ferrer et al., 2020b) in the sample and the curve is almost logarithmic. In fact, the time evolution of the “lag-phase” is also of interest for the characterization of some amyloids (i.e., MSA-derived synuclein vs. PD-derived synuclein) with different seeding as well as propagative properties (i.e., Shahnawaz et al., 2020). Additionally, in most studies the molecular events that occur in this “lag-phase” of amyloid formation are not fully ascertained using classical analytical methods. However, LOC devices and microdroplet formation were very useful in describing, in greater detail, the sequential events during this fibril formation for different amyloids (i.e., Aβ; Arosio et al., 2014), silk fibroin, β-lactoglobulin, lysozyme (Toprakcioglu et al., 2019), human islet amyloid polypeptide (Marek et al., 2010), and sickle hemoglobin (Ferrone et al., 1985). In fact, the events that occur during this lag-phase are of special interest in the effort to understand amyloid dynamics during neurodegeneration (Orru et al., 2015; Masujin et al., 2016).

In neurodegeneration, although with some discrepancy, it is well-established that lower aggregative amyloid species are more relevant species (i.e., toxic) compared to large amyloid species in different diseases [i.e., for Aβ (Deshpande et al., 2006; Hepler et al., 2006) and α-synuclein (Conway et al., 2000; Winner et al., 2011; Chen and Cremades, 2018; Prots et al., 2018)]. More relevantly, researchers aim to determine the properties of the “propagative seeds” or “propagons” for particular amyloids. This is a challenging question, especially for “*prion-like*” proteins (Aguzzi and Lakkaraju, 2016). Over time, classical bulk methods have been modified to match those that include an amplification step. For example, using a new method, Arosio et al. (2014) detected Aβ propagons in a “lag-phase” of aggregation by sample filtration during the phase, followed by an amplification method with fresh Aβ monomer. This was followed by the quantification of the original propagon concentration using a calibration curve based on controlled seed concentration (Arosio et al., 2014). Using this method, the authors improved by two orders of magnitude the bulk technical approaches to allow the concentration of fibrillar Aβ (Arosio et al., 2014). However, the recent development of “digital microfluidics” which combines the use of microfluidics and high-throughput biological assays (Guo et al., 2012) has helped researchers to develop a digital amyloid quantitative assay (d-AQuA) aimed at allowing absolute quantification of single replicative units, the “propagons,” in the proof-of-concept manuscript of insulin (Pfammatter et al., 2017). In fact, the authors used a dilution method of nanodroplets with picolitres of volume containing (or not) “propagon” molecules that were further evaluated using ThT staining, following a Poisson distribution probabilistic model (Pfammatter et al., 2017). This method, although not developed for other amyloids, is faster than currently available methods (e.g., microplate assays) and will be of relevance for fast diagnosis of the presence of “pathological-seeds” for different PMDs. Parallel to these approaches, other groups have developed more automatic methods such as the microchannel-connected multiwell plate (μCHAMP) device (Park et al., 2016) that uses microdroplet formation and microfluidic transport to 96-well plates. The amount of Aβ (at a range of ≈ 10 pg/mL) is detected using a droplet-based magnetic bead immunoassay (Park et al., 2016). Thus, LOC devices are of relevance not only for the reduced sample volume required, but also for automatization and high-throughput assays of amyloid detection. In addition, recent reports illustrate the relevance of the use of aggregation-induced emission (AIE) dyes as a preferential strategy to identify protein fibrillogenesis, particularly the light up characteristic associated with binding events during the aggregation process. These dyes feature high emission efficiency in the aggregate state, strong photo-stability, and excellent biocompatibility (Wurthner, 2020). Examples of their use can be found in the analysis of insulin (Hong et al., 2012; Huang Q. et al., 2017) and of β-amyloid aggregation (Zhang J. D. et al., 2016; Fu et al., 2019; Yang et al., 2019). Readers may find a general survey of these techniques in Wurthner (2020).

## LOC as Tool to Analyze Cell-To-Cell Transport and Behavior of Amyloids

As indicated above, studies of seeding and spreading of different amyloids *in vitro* must be complemented by complex cellular systems mimicking *in vivo* situation as well as animal experimentation. Indeed, several groups have developed *in vitro* cellular models to monitor seeding properties and their associated effects in cell survival. However, for some amyloids (e.g., tau) their overexpression in cells resists aggregation instead of hyperphosphorylation, and additional methods are needed to increase their intracellular content, avoiding phosphorylation. One of the alternatives is a commercially available BioPORTER QuikEase Protein Delivery Kit which can deliver active proteins intracellularly. This method was used by Guo and Lee (2011) to demonstrate that the seeding of normal tau induced by pathological tau led to the formation of NFL-like structures in treated cells (Guo and Lee, 2011). In other approaches, cell lines overexpressing mutated protein with aggregative properties fused with a flag (fluorescence) sequence (i.e., P301L-V5; Xu et al., 2016) have been used for high-throughput assays of tau aggregation. In the presence of an aggregating amyloid species (e.g., tau) the aggregation process can be followed by analyzing the emitted fluorescence. Some of these approaches have also included FRET methods (Shin et al., 2019). These cellular “biosensors,” mainly developed in HEK293 or H4 cells, have been adapted for fluorescence detection of other amyloids such as α-synuclein (Prusiner et al., 2015; Holmes and Diamond, 2017). In fact, these methods are already being supported by the use of ultra-resolution confocal microscopy (Kaminski and Kaminski Schierle, 2016) to monitor the amyloid seeding and aggregation processes in cell cultures at the nanoscale level. Today the use of these biosensors is mainly focused on drug screening. However, in a putative scenario the use of these cells in LOCs could be of relevance in determining key factors involved in the seeding process of amyloids (e.g., the relevance of a specific receptor in the process).

**FIGURE 5:**
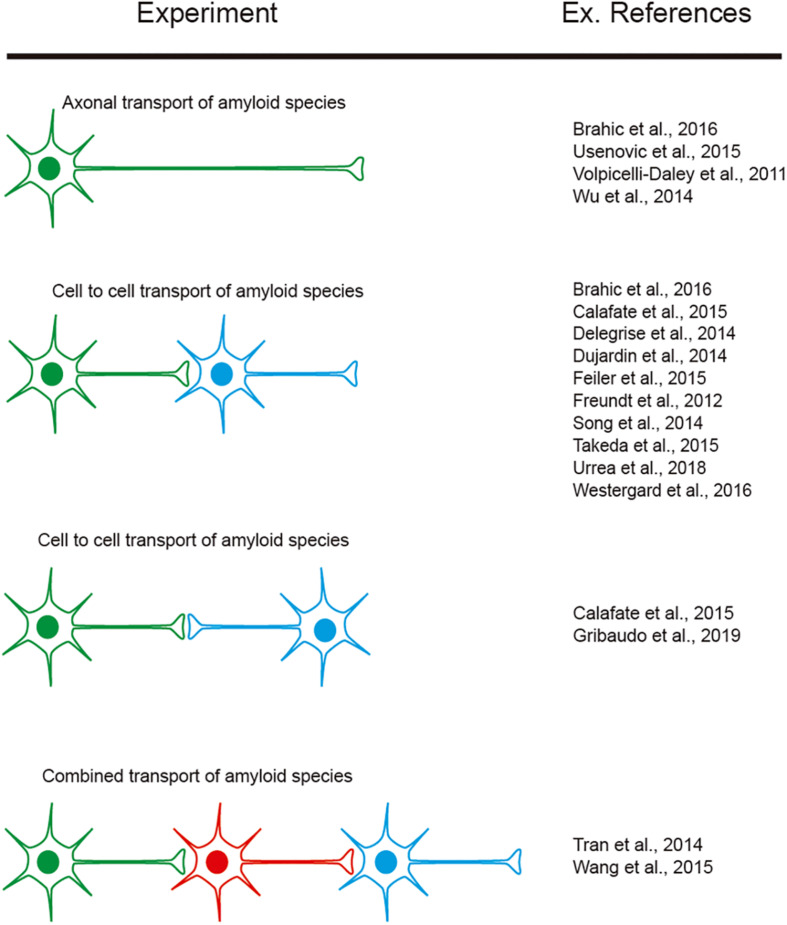
Examples of experiments designed to explore amyloid transport in axons as well as cell-to-cell transmission. The different colors of the illustrated cells represent the cells cultured in different reservoirs of compartmentalized LOC devices and their orientation. In addition, some references are included as examples of their exponential use in neurodegenerative neuroscience.

However, as indicated, researchers aimed to determine cell-to-cell mechanisms implicated in amyloid seeding and spreading (see introduction). In this respect, several studies reported using LOC devices, with two or more consecutive chambers: Aβ (i.e., Deleglise et al., 2014; Song et al., 2014), α-synuclein (i.e., Volpicelli-Daley et al., 2011; Freundt et al., 2012; Brahic et al., 2016; Urrea et al., 2018; Wang et al., 2018), tau (i.e., Wu et al., 2013; Dujardin et al., 2014; Calafate et al., 2015; Takeda et al., 2015; Usenovic et al., 2015; Congdon et al., 2016; Polanco et al., 2018), TDP-43 (i.e., Feiler et al., 2015), and dipeptide repeat proteins (DPRs) of the *C9orf72* gene product associated with ALS and frontotemporal dementia (FTD). Westergard et al. (2016) were able to perform cell-to-cell transmission by navigating intracellularly along axons allowing seeding and propagation of the amyloid (reviewed in Urrea et al., 2018; Peng et al., 2020; Uemura et al., 2020). These studies reported the sequential transport of the “pathogenic seeds” as free seeds or in exosomes between different cell populations cultured in different chambers (Figure 5). In these experiments a differential volume between reservoirs is established to avoid diffusion transfer by the media between reservoirs. In fact, different LOCs have been designed for specific studies (e.g., co-culture of microglia, astroglia, and neurons (3D hNeuroGliAD) (Park et al., 2018). More relevantly, in several studies neurons derived from IPSc or neuronal progenitors are included to mimic specific neurodegenerative diseases (i.e., Choi et al., 2014; Ruiz et al., 2014; Park et al., 2018). These LOC approaches help researchers to ascertain the role of non-neuronal cells in the seeding and propagation process for particular amyloids (e.g., Aβ roles of microglia reactivity and migration (Cho et al., 2013; Park et al., 2018) and the role of astrocytes in α-synuclein seeding (Cavaliere et al., 2017). However, the emerging role of oligodendrocytes as non-neighboring cells during some amyloid transmission [i.e., α-synuclein (Tu et al., 1998; Uemura et al., 2019), tau (Ferrer et al., 2019, 2020a) see also (Ferrer, 2018) for recent review] has yet to be described in LOC devices. This is in contrast to the different LOC devices aimed at exploring myelination (Park et al., 2009; Yang et al., 2012; Lee et al., 2016). Further studies will help neuroscientists to determine their putative role in PMD.

## LOC as a Tool to Analyze the Neural Consequences of Amyloid Transmission

From the early development of the PDMS compartmentalized LOC, several groups combined electrical and optical measurements of neuronal activity with its compartmentalization (among others, Morales et al., 2008; Gladkov et al., 2017; Lassus et al., 2018; Lopes et al., 2018; Moutaux et al., 2018). And in some studies these LOCs devices were used as platforms to determine drug screening and cytotoxicity (i.e., Meissner et al., 2011; Du et al., 2016). As noted, the seeding and spreading of different amyloids is associated with an increase in progressive cell death. This was determined in classical cell cultures in single chambers but particularly in LOC with different chambers: (i.e., AβDeleglise et al., 2014; Ruiz et al., 2014; Li et al., 2017), α-synuclein (i.e., Tran H. T. et al., 2014; Prots et al., 2018; Gribaudo et al., 2019) and Tau (Wu et al., 2016) by direct exposure of cultured neurons to the pathogenic form of the amyloid. Cytotoxicity was measured with biochemical methods, multielectrode arrays (MEA), or changes in calcium transients [i.e., with Fluo4-AM or genetically encoded calcium indicators (GECIs)]. In this respect, several authors included microflows in 3D-derived cultures in LOC devices to determine the effects of exposure to amyloids. For example, Choi et al. (2013) developed a microfluidic platform capable of generating a gradient of Aβ oligomeric assemblies within microchannels to investigate their neurotoxicity. Two years later, Park et al. (2015) developed a pioneering microfluidic chip containing 3D-neurospheroids by providing an interstitial constant fluid flow. Using this 3D platform, the effect of the fluid flow on the neural proliferation, differentiation, and survival was investigated. The authors compared their results with similar Aβ treatments under static conditions, and the main conclusion was that treatment with Aβ under interstitial flow is more deleterious and largely reduces the viability of neural aggregates (Teller et al., 2015).

**FIGURE 6:**
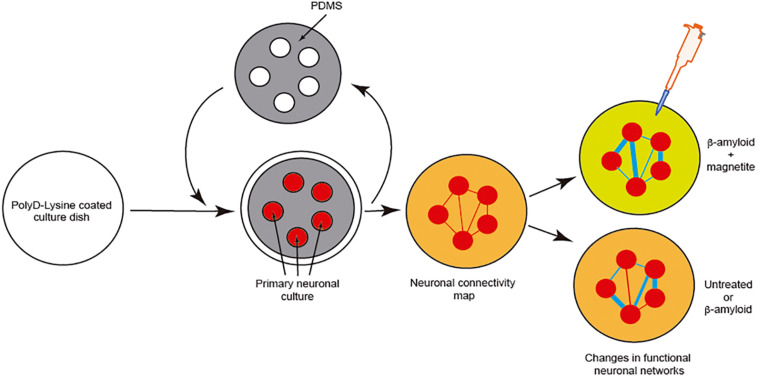
Example of the analysis of neuronal network changes mediated by amyloids (Aβ). We describe the experiment developed by Teller et al. (2015). In the experiment, the authors generated clusters of cells further interconnected in a network like the Atomium attraction in Brussels. After treatment with Aβ alone or Aβ-magnetite, the changes in the neural network were analyzed using NetCal^TM^ software.

However, although effects of the pathogenic seeds have been evaluated in numerous manuscripts, little attention has been paid to elucidating the putative changes in neural networks during seeding and cell-to-cell progression between different areas. Due to the relevance of this issue, LOCs could be a relevant tool to ascertain this progressive neurodegeneration between different cell populations. This is of particular relevance for drug discovery, if our final goal is to block amyloid seeding, aggregation and progression. For most cases, a putative drug treatment (i.e., an inhibiting peptide) could be useful *in silico*, and probably non-cytotoxic in yeast, worm, fruit flies, or mammalian cells, but it could carry collateral effects in modifying the function of neuronal networks. To solve this, in our opinion, we need first to understand the changes induced by pathogenic amyloid during the seeding and aggregation process from both the cellular viewpoint and a system-wide perspective. One example can be seen in a pioneer study developed by Teller et al., in which primary cortical neurons were confined into groups using a homemade PDMS mask (Figure 6). In the study, the authors determined the synaptic activity and the development of neuronal networks by using calcium fluorescence probes (Fluo4-AM) and the analysis of the intracellular calcium changes to determine firing rate, coordination between different neurons, and the appearance and maturation of different connectivity “hubs” in the culture. After several days in culture to allow the maturation of connections between these neuronal groups, the authors incubated the culture with different aggregated forms of Aβ (with magnetite). Changes in neural network activity and the correlated neuronal interactions after the treatments were evaluated, and the effects of Aβ treatment in the destabilization of the generated network was determined in detail. In order to analyze this the authors used homemade calcium fluorescence analysis software NetCal^TM^ (Orlandi et al., 2014; Teller et al., 2015; Figure 6). The data were also corroborated in our laboratory using mic2net software (Smedler et al., 2014). Thus, the group developed an *in vivo*-like platform for drug screening in the presence of progressive amyloid generation. Today the use of GECIs (i.e., GCaMP6) instead of classical fluorescence reporters (Fluo4-AM or Fluo8-AM) of neuronal activity and different analysis methods allow us to analyze the network activity of the same culture or neuronal network for longer time periods (Chen et al., 2013). These experiments will help researchers working in drug discovery to avoid the seeding and progression of the amyloids without perturbing neuronal activity.

## Concluding Remarks

In this review we have tried to summarize only a part of the state of the art concerning the use of some microfluidics and LOC devices in amyloid or “*prion-like*” seeding, aggregation, and cell-to-cell transmission research. Unfortunately, for prion infection other strategies would be more appropriate due to the time needed to develop a reliable prion infection. In fact, our lab studied the putative prion formation in IPSc cultured in 2D cultures for more than 150 days with negative results (Matamoros-Angles et al., 2018). Considering this, most probably a 3D culture is needed to develop prion infection, while LOCs will help us in other experimental situations. In this review, we have focused, on a practical basis, on those aspects that we consider relevant for biomedical researchers. In fact, most of the new techniques and proof-of-concept experiments involving aggregation, protein-protein interaction, and analyte detection are not fully described in this review, since, in some cases, they are not yet widely accepted by researchers. We refer the reader to some of the references of this review for additional information. In fact, here we have focused on two types of LOC that could be of interest and which are commonly used in laboratories. For example, micro/nanodroplets LOCs, compared to conventional microtiter plate assays, are an attractive platform for high-throughput studies. LOC devices derived from the compartmentalized culture of neurons or glial cells can help answer key questions in determining cell-specific differences in various disorders, revealing the participation of different cell types, uncovering the differing behaviors of the different “strains” of amyloid, etc. In addition, emerging technologies such as the use of graphene and other materials such as electrochemical and optochemical “biosensors” offer new opportunities to determine amyloid seeding and aggregation in more detail. As indicated, the bioengineering approaches have evolved in recent years, and today, some of them are tools for neurobiological laboratories, as an alternative to unnecessary animal experimentation following the 3Rs of biological research. Although we cannot assume that the use of LOC and microfluidics in neuroscience will follow Moore’s law of semiconductors, which suggests doubling of semiconductor numbers in a microprocessor every year, we believe that their use helps and will continue to help neuroscientists. Combined efforts from different disciplines—bioengineering, neurobiology, and clinical practice – in the face of new challenges will be mandatory for future biomedical research.

## Author Contributions

JR and IF: drafting of manuscript, study conception and design, analysis and interpretation of data, critical revision, and final approval of the version to be published. Both authors contributed to the article and approved the submitted version.

## Conflict of Interest

The authors declare that the research was conducted in the absence of any commercial or financial relationships that could be construed as a potential conflict of interest.
